# Detailed Association Between Electrocardiogram Abnormality and Primary Myocardial Fibrosis

**DOI:** 10.1002/clc.70237

**Published:** 2025-12-15

**Authors:** Yu Nomoto, Naoya Kataoka, Teruhiko Imamura

**Affiliations:** ^1^ Second Department of Internal Medicine University of Toyama Toyama Japan

**Keywords:** J‐wave syndrome, sudden cardiac death, ventricular fibrillation

To the Editor,

Sudden cardiac death (SCD) remains an unresolved issue even in the contemporary era of implantable cardioverter‐defibrillator therapy (ICD), largely because of the difficulty in accurate risk prediction. The present study sought to identify clinical features of SCD victims with non‐ischemic myocardial fibrosis [1]. The authors found that fragmented QRS complexes or a history of transient loss of consciousness (TLOC) were major characteristics of such patients. While this is an important contribution, several concerns arise.

Our group previously reported that the presence of a J‐wave, reflecting repolarization abnormalities—similar to QT prolongation and T‐wave inversion—was associated with ventricular fibrillation as the terminal electrocardiographic event [2]. Conversely, a fragmented QRS represents an abnormality of depolarization rather than repolarization. In the present study, were the terminal electrocardiograms (ECGs) of SCD victims with fragmented QRS more commonly asystole or pulseless electrical activity rather than ventricular fibrillation? How do the authors interpret the relationship between different types of polarization abnormalities and the final rhythm observed? Clarifying this relationship would be crucial for risk stratification and further pathophysiological understanding.

Another concern lies in the limited availability of clinical data. Only 28% of victims had ECGs available, and merely 11% had both ECG and medical history [1]. Such a restricted dataset raises the possibility of selection bias and questions the generalizability of the findings. Moreover, TLOC, although frequent, is not necessarily of cardiac origin. Without detailed adjudication, including differentiation from neurological or reflex‐mediated causes, the specificity of this marker as an indicator of impending SCD remains uncertain.

The pathophysiological link between fragmented QRS and myocardial fibrosis was also insufficiently addressed [1]. Prior studies have demonstrated that fragmented QRS may reflect impaired conduction due to localized scar or interstitial fibrosis, and that it correlates with abnormal global longitudinal strain [3]. Moreover, fragmented QRS is also associated with arrhythmic events in other entities such as Brugada syndrome. However, whether fragmented QRS in the setting of primary myocardial fibrosis is merely a surrogate of structural remodeling or a direct arrhythmogenic substrate remains unanswered.

It should also be emphasized that the features highlighted in this study—fragmented QRS and TLOC—are neither novel nor specific to primary myocardial fibrosis. Given that ICD implantation cannot be justified in all individuals with these findings alone, we believe these markers should be integrated into a broader, multi‐modal risk stratification strategy. Cardiac magnetic resonance imaging to detect diffuse or patchy fibrosis, genetic testing for inherited cardiomyopathies, and modern arrhythmia monitoring may offer a more refined approach to identifying high‐risk individuals.

## Funding

The authors received no specific funding for this work.

## Ethics Statement

The authors have nothing to report.

## Consent

The authors have nothing to report.

## Conflicts of Interest

The authors declare no conflicts of interest.

## Data Availability

The authors have nothing to report.

## References

[1] H. Silvola , L. Pakanen , L. Holmström , et al., “Primordial Symptoms and ECG Among Sudden Cardiac Death Victims Due to Primary Myocardial Fibrosis,” Clinical Cardiology 48 (2025): e70057. 40590619 10.1002/clc.70057 PMC12210387

[2] N. Kataoka , T. Imamura , K. Uchida , T. Koi , and K. Kinugawa , “Significance of J Waves in Unexplained Ventricular Fibrillation Among Elderly Populations With Various Comorbidities,” Heart Rhythm O2 5 (2024): 417–420. 38984359 10.1016/j.hroo.2024.05.004 PMC11228269

[3] M. R. Dehghani , A. Rostamzadeh , A. Abbasnezhad , A. Shariati , S. Nejatisafa , and Y. Rezaei , “Fragmented QRS and Subclinical Left Ventricular Dysfunction in Individuals With Preserved Ejection Fraction: A Speckle‐Tracking Echocardiographic Study,” Journal of Arrhythmia 36 (2020): 335–340. 32256883 10.1002/joa3.12284 PMC7132185

